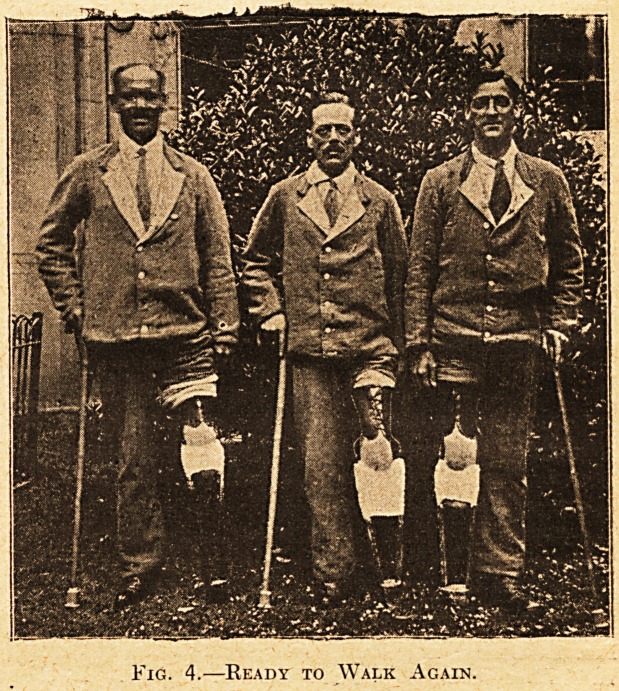# As Made at the Pavilion General Hospital, Brighton

**Published:** 1918-07-13

**Authors:** G. T. K. Maurice

**Affiliations:** Colonel A.M.S., O.C. Pavilion General Hospital.


### TEMPORARY ARTIFICIAL LEGS

### As Made at the Pavilion General Hospital, Brighton.

### II. Above-the-Knee Legs—(*cont.*).

The Making of the Cast.

The patient stands on a stool on his good leg. The stool is beside the table. On the table is a Windsor chair with a cushion on the seat, on which the man supports himself. The stump is on the side away from the table, so that the operator can easily get at it. Below the stump is a second stool, 1 inch lower than that on which the man is standing. The reason of this difference is that the man tends to droop over towards the stump, so if the stools be equal the artificial leg is short. A mackintosh sheet and some sheets of old newspaper are fastened so as to protect the patient's clothes from splashes of plaister. The stump is naked.

Fig. 1.—Stages and Parts of a Leg for Thigh Stump. A. Rubber pad. B. Base block. C. Spline. D. The mould has been made and fixed to splines. E. The hollows have been filled and the bandages to finish applied. F. The final trimming and binding is done, the strut is in the leg completed. K. The hook—hidden in E. and F.

The operator now greases the stump with vaseline, puts patches of oil silk on very hairy parts, and fills any hollows or spaces or depressed scars with cotton-wool. This sticks to the vaseline, so does not fall off.

Beginning at the lower part of the stump, but never covering the end of it, and working upwards and applying them flatly but quite loosely, the operator winds plaister-of-Paris bandages round and round the stump, cutting them whenever a turn would be necessary. The bandages are dipped in water before being used. When five or six layers have been applied—the number depends on the amount of plaister in the bandages and is a matter of judgment—the wooden frame is brought to the operator and adjusted to the position it is to be in. The splines are on each side of the stump, and the all-important point is that they shall be parallel to the sides of the median latero-vertical plane, so that the man when on his leg shall neither stand on his heel nor his toes so to say. This is especially important to remember when dealing with flexed stumps. The vertical axis of many stumps is diverted outwards. If this be so, the frame has to be set so that the vertical axis of the stump and the vertical axis of the frame meet at an obtuse angle. If not, the base block will be very far from the other foot, and the man will be "walking wide" as we say here.

The two splines when in position will be in part touching the plaister bucket, in part away from it. Of each the outer aspect as regards the stump is slightly grooved with a file at the upper part. Plaister bandages are taken again, and the splines

Fig. 2.—Operator making Above-knee-stump Leg. (Note unequal stools and application of figure-of-eight strips round splines.)

and the bucket fixed together by figure-of-eight bandaging—the inner spline is fixed first. An end of bandage is laid on the anterior aspect of the bucket, the roll is passed under the spline, over the spline, and under again, and passed to the middle line or just over the back of the bucket. The bandage is cut, the end of the strip plaistered

The previous article appeared on July 6, p. 303.

**Temporary Artificial Legs—(*continued*).**

down, and a fresh figure-of-eight applied. It requires five to eight such strips round each spline.

The bucket is now slipped off the stump and the leg put aside to dry and await completion. The interior of the bucket is a mould of the stump. The ischial tuberosity is included in the mould. It is chiefly this tuberosity that takes the pressure and transmits the patient's weight to the leg.

The next stage is to pack the hollows and spaces between the thin mould and the splines with cotton-wool soaked in plaister of Paris to get the necessary thickness and strength. This is done by pressing in plugs of wool, small at first and larger later, and moulding with fingers dipped into plaister. By this process the splines are firmly fixed and enough substance built up to give the requisite strength. During this process "the hook" is fixed in its position.

THE HOOK.

"The hook" (illustrated in Article 1) is one of the attachments for "the harness." It is made

FIG 3.—SHOWING THE HARNESS.

of short lengths of metal cut from long strips of $\frac{3}{4}$ inch wide $\frac{3}{32}$ inch thick iron. One piece is cut $5\frac{1}{2}$ inches long, a second 4 inches long. This second piece is divided longitudinally, and one of the pieces so obtained bent at an obtuse angle $\frac{3}{4}$ of an inch from one end, and again at an obtuse angle $\frac{3}{4}$ of an inch beyond this bend, so as to form a flat "Z" shaped piece. The shorter end of the "Z" is drilled. The longer and broader straight piece is drilled also between $1\frac{1}{4}$ and $1\frac{1}{2}$ inches from one end. The "Z" shaped piece is riveted to the straight piece and thus a hook is formed. The longer portion of the straight piece projects beyond the point of the hook. The straight piece is now incorporated in the plaister, the upper end reaching the top of the outer spline, the end of the hook pointing downwards, and secured by turns of bandage. The

FIG. 4.—READY TO WALK AGAIN.

position of the hook is behind and against the outer spline. It projects about $\frac{1}{2}$ inch.

A few more turns of plaister bandage are passed round the whole cast, and the apparatus is finished off by a little plaister-work and smoothing done by the operator's hands. The result is that the splines are buried in plaister at the upper part, the hollows behind and in front of them have disappeared, and smooth curves run right round the whole mould, which is especially strong on the outside and inside.

ADJUSTING THE MOULD.

The mould is put aside to dry. Next day the man comes for final adjustment. The mould is slipped on his stump and marked with pencil to show how much of the upper and lower edges may be cut away. This cutting away is done with strong scissors till the upper edges are nicely tidy and comfortable to the man. A good deal may be cut off in front, but at the back very little. The lower end is cut away till the stump projects comfortably. When the man is quite comfortable the leg is removed and the upper edge bound with a long strip of plaister bandage folded, and fastened by little pieces of plaister bandage about 4 inches long and put over piece by piece from inside to outside.

The strut is now put in position. This is a piece of wood (the grip of an unserviceable crutch will often serve) placed in between the splines and fixed by screws that go through the splines and into the

(*Continued on p.* 322.)

(*To be continued.*)

### Temporary Artificial Legs.

(*Continued from p.* 324.)

ends of the strut. It and the hook give purchase to "the harness."

The harness is made of webbing 2 inches wide. It consists of four pieces. One piece has a buckle at one end, and goes under the strut and over the shoulder of the side away from the stump and buckles in front between the waist and the strut. One piece goes round the waist like a belt. A third piece has a runner loop at one end, through which the waist-piece goes, and a buckle at the other. A fourth piece has a "D" at one end and an end to go into the buckle of the runner loop. The "D" is hitched on the hook and the length adjusted by the buckle. A stump sock is put on the stump, the leg put on; the man is helped for a turn or two round the room, and then is able to go on his way, nervous perhaps, but rejoicing.

G. T. K. Maurice, Colonel A. M. S.,
O.C., Pavilion General Hospital.

## Figures and Tables

**Fig. 1. f1:**
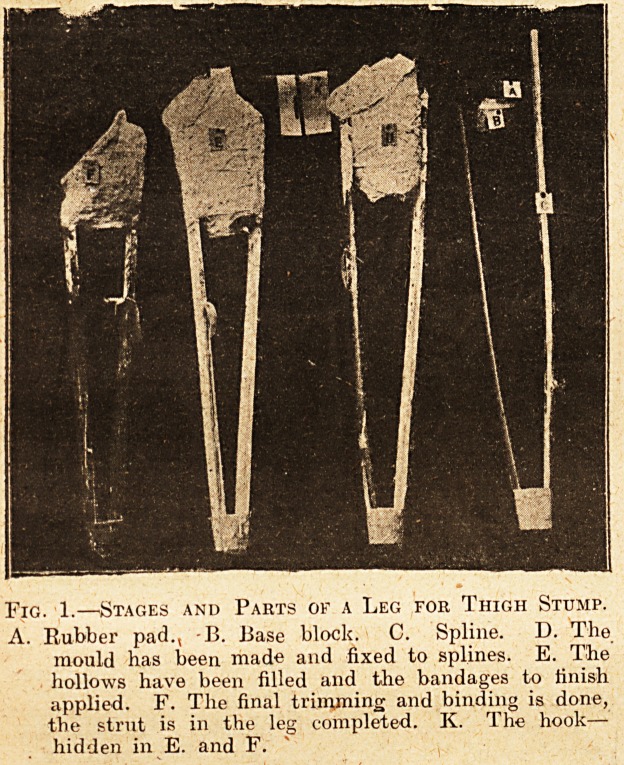


**Fig. 2. f2:**
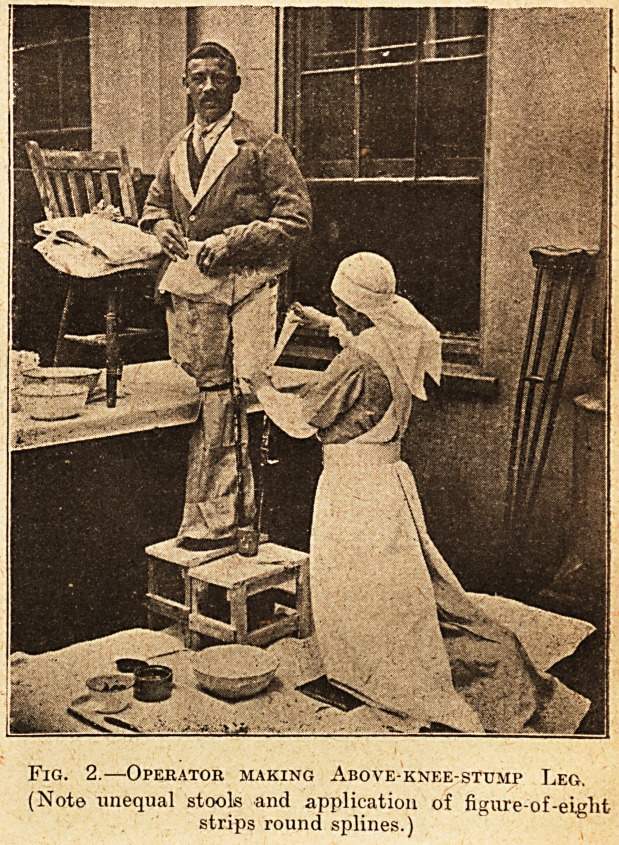


**Fig 3. f3:**
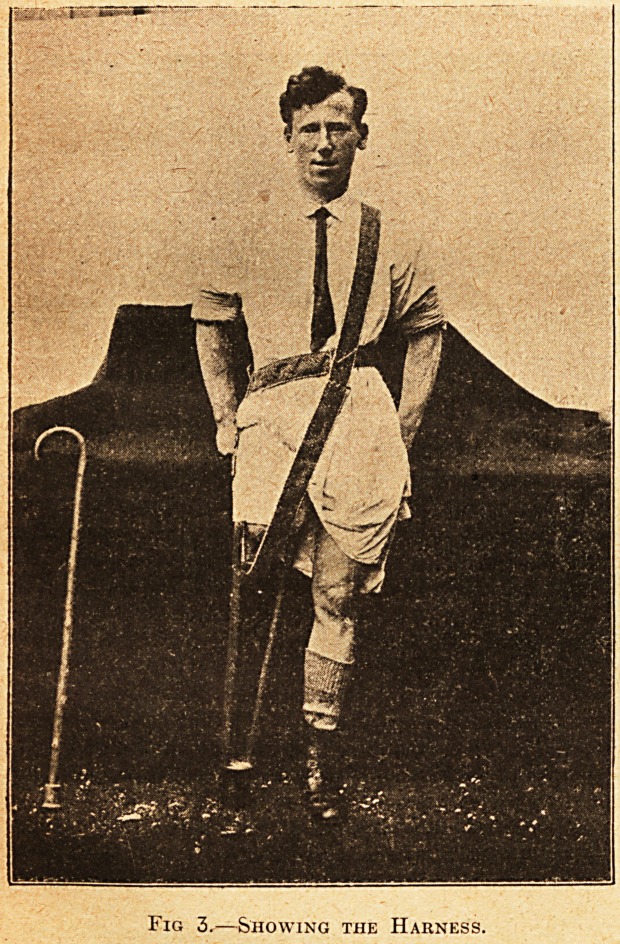


**Fig. 4. f4:**